# Targeting TRPV1 to relieve motion sickness symptoms in mice by electroacupuncture and gene deletion

**DOI:** 10.1038/s41598-018-23793-6

**Published:** 2018-07-09

**Authors:** Chanya Inprasit, Yi-Wen Lin, Chun-Ping Huang, Shu-Yih Wu, Ching-Liang Hsieh

**Affiliations:** 1 College of Chinese Medicine, Graduate Institute of Acupuncture Science International Master Program, China Medical University, Taichung, 404 Taiwan; 2 Chinese Medicine Research Center, China Medical University, Taichung, 404 Taiwan; 3 College of Chinese Medicine, Graduate Institute of Acupuncture Science, China Medical University, Taichung, 404 Taiwan; 4 Department of Rehabilitation Medicine, Mackay Memorial Hospital, Taipei, Taiwan; 5 College of Chinese Medicine, Graduate Institute of Integrated Medicine, China Medical University, Taichung, 404 Taiwan; 6 Department of Chinese Medicine, China Medical University Hospital, Taichung, 404 Taiwan

## Abstract

Motion sickness (MS) is an acute disorder that occurs in healthy individuals worldwide regardless of gender, age, or ethnicity. Our study used a mouse model to rule out the effects of any psychological factors related to MS and EA. Subjects were randomly separated into four groups, namely the control group (Con), motion sickness inducing group (MS), mentioning sickness inducing with electroacupuncture treatment group (EA) and motion sickness inducing only in TRPV1 knockout mice group (TRPV1^−/−^). The consumption of kaolin, a non-nutrient substance, was measured as a behavior observed response of an emetic reflex in a murine model. This behavior is referred to as pica behavior. Our results showed that pica behavior was observed in the MS group. Moreover, kaolin consumption in the EA group decreased to the average baseline of the control group. A similar result was observed in TRPV1 null mice. We also observed an increase of TRPV1 and related molecules in the thalamus, hypothalamic and brain stem after MS stimulation and a significant decrease in the EA and TRPV1 null groups. This is the first study to demonstrate that TRPV1 pathways are possibly associated with mechanisms of MS, and can be attended through EA or TRPV1 genetic manipulation.

## Introduction

Motion sickness (MS) occurs in healthy individuals worldwide regardless of gender, age, or ethnicity. MS is well known to be triggered by frequently changing movements, inducing nausea, vomiting, dizziness, drowsiness, and fatigue. Moreover, MS can be initiated without movement; this is known as pseudo-MS and can occur, for example, when playing a game, watching a movie, or running on a treadmill^1^. Various preventive techniques and treatments for MS have been discovered, including behavior change before and during MS-inducing situations, ginger consumption, acupressure, and acupuncture. All MS responses result from a conflict between neural mismatch signal inputs and neural stores in the cerebellum^2–5^. Approximately 5–10% of people are highly susceptible to MS, and the remainder show moderate susceptibility^1^. Many factors provoke strong reactions to MS, and these may be heightened at the beginning of the menstrual cycle or during pregnancy in women^6,7^. The sight and smell of food or drinking alcohol can also provoke MS responses^8^. The most common Western medical treatments for MS are anti-histamines, anti-cholinergic agents, and anti-noradrenaline^9^. Pica behavior, which is the consumption of non-nutritive substances, was found to be a good measure of the MS response in rats^10,11^. Pica behavior is thus an index of MS in rodents, basically, the gastrointestinal responses^12^. The input signals from vestibular organs in the inner ear and somatosensory cascade to the thalamus, the center of sensory information, before flowing to other parts of the brain, which associate with MS responses, including the hypothalamic and brain stem regions. Additionally, the oculomotor system signal pathway initiates in the brain stem, and cooperates with the vestibular system for positional recognition responses. Furthermore, the hypothalamus was indicated in the modulation of histamine receptor mRNA expression in the paraventricular and vestibular nuclei. The upstream area and nucleus of a solitary tract were observed in the brain stem^13^. The mismatch signals from these three brain areas are important in the exploration of the TRPV1 expression in MS in mice.

There are six members in the transient receptor potential vanilloid subfamily, which respond to neurotransmitters and propel signals to the subsequent neurons. Among the 6 members, the transient receptor potential cation channel subfamily V member 1 (TRPV1) was the first to be isolated and is the most special, being characterized as a homotetrameric non-selective calcium-permeable cation channel^14–18^. Many reports have described the distribution of TRPV1 in widely expressed brain areas, including the thalamus, hypothalamus, hippocampus, amygdala, cerebellum, and dorsal root ganglia^17,19,20^. It is well known that TRPV1 plays an important role both in neuropathic and nociceptive pain as well as in inflammation, due to the fact that low pH (<6.0) and high temperature (>43 °C) are capable of activating TRPV1 channels^21^. Furthermore, anxiety, stress, depressed behavior, chronic cough, irritable bowel syndrome, and urge incontinence have been found to be associated with TRPV1^22^. When TRPV1 is activated, ion channels allow calcium ion influx, causing membrane depolarization. Intracellular signals then cascade to proteins, including protein kinases, mitogen-activated protein kinase (MAPK), and cyclic AMP-response element binding protein (CREB).

MAPK signaling can mediate signals between the nucleus and cell surface and is involved in several extracellular stimuli, including human diseases, stress, heat, inflammation, and cancer^23^. There are three major groups of MAPKs, including extracellular signal-regulated kinase (ERK), c-Jun N–terminal protein kinase (JNK), and p38 kinase. Furthermore, there exist diversely related pathways, such as phosphatidylinositide-3 kinase (PI3K), v-akt murine thymoma viral oncogene (AKT), mammalian target of rapamycin (mTOR), and nuclear factor-kappa B (NFκB). In addition, some aspects of MAPK signaling associate with the calcium/calmodulin-dependent protein kinase and CREB in neuronal pathways^24^.

Acupuncture has been practiced for over 3,000 years and recommended by the World Health Organization. Acupuncture is becoming more widely accepted by foreign practitioners and universally by patients, and the number of papers on acupuncture treatment for functional gastrointestinal disorders, including nausea and vomiting under MS conditions, has increased as of late^25^. Evidence-based studies have suggested that electroacupuncture (EA) can be used to treat learning and memory impairments in rats with cerebral ischemia-reperfusion injury^26^ or epilepsy^27^ and to control body weight^28^ and pain^29–32^. Nei Guan, also known as acupoint pericardium 6 (PC6), is the most commonly important acupressure point, especially for nausea and vomiting^33^, and can extend the time to onset of nausea, even though its function remains unknown^34^. In the present study, we hypothesized that the EA treatment would reduce the symptoms of MS through the TRPV1 pathway.

## Results

**Figure 1:**
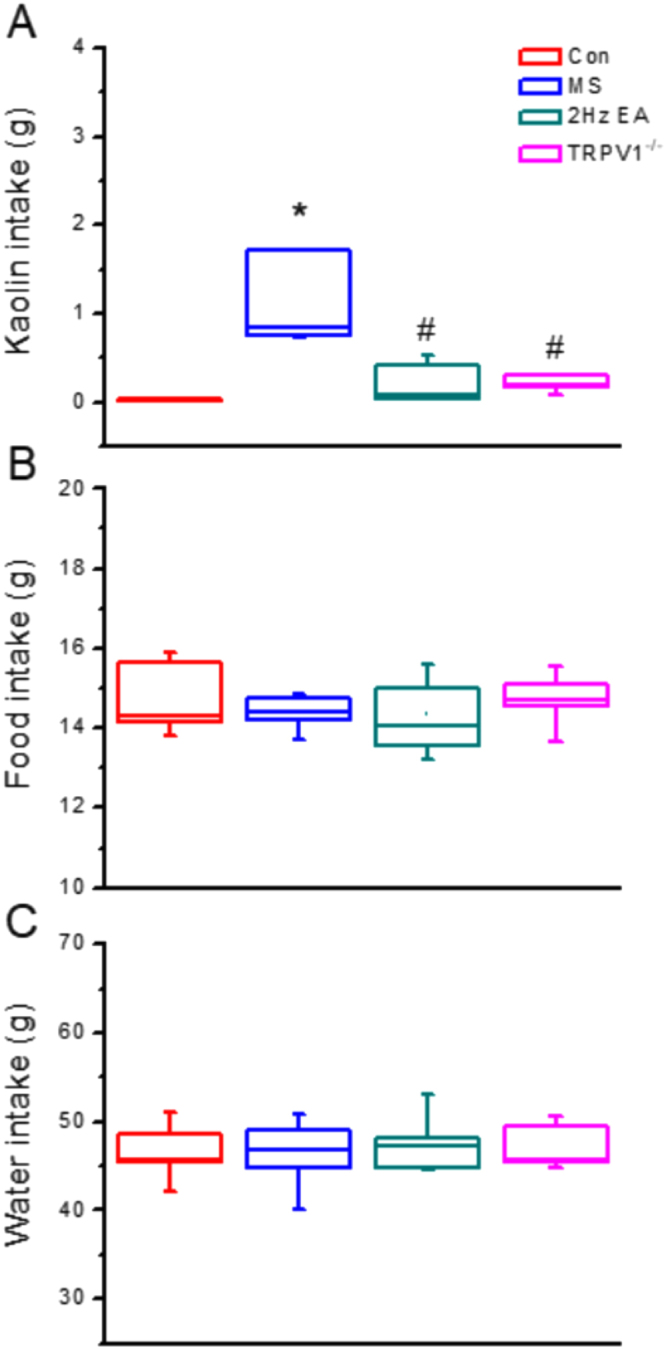
Daily kaolin, food, and water consumption of mice in four groups: Con, MS, EA, and TRPV1^−/−^. Kaolin consumption in the MS group was significantly greater than that in the other groups (^*^p < 0.05, compared with the Con group). In contrast, it was significantly decreased in the EA group and in the TRPV1^−/−^ group after EA treatment, which lacked the TRPV1 gene (^#^*p* < 0.05, compared with the MS group). Con = Control; MS = Motion sickness; EA = 2-Hz electroacupuncture; TRPV1^−/−^, TRPV1 gene deletion. ^*^p < 0.05 vs. Con. ^#^*p* < 0.05 vs. MS group.

MS in this mouse model was assessed by measuring kaolin consumption during the experimental period. Data of 24 mice were analyzed and are presented in Fig. 1. Throughout the experimental period, we observed a significantly increased kaolin consumption in the MS group (Fig. 1A; 1.13 ± 0.19, *p* < 0.05) compared with that in the control group. In contrast, kaolin consumption was significantly decreased in the EA group (Fig. 1A; 0.22 ± 0.09, *p* < 0.05) and even more so in the TRPV1^−/−^ group (Fig. 1A; 0.22 ± 0.03, p < 0.05) compared with that in the MS group. To examine behavior change in the animals, we also observed food and water consumption. We observed no significant difference in food (Fig. 1B; *p* > 0.05) and water (Fig. 1C; *p* > 0.05) consumption among the control, MS, EA, and TRPV1^−/−^ groups throughout the experimental period.

**Figure 2:**
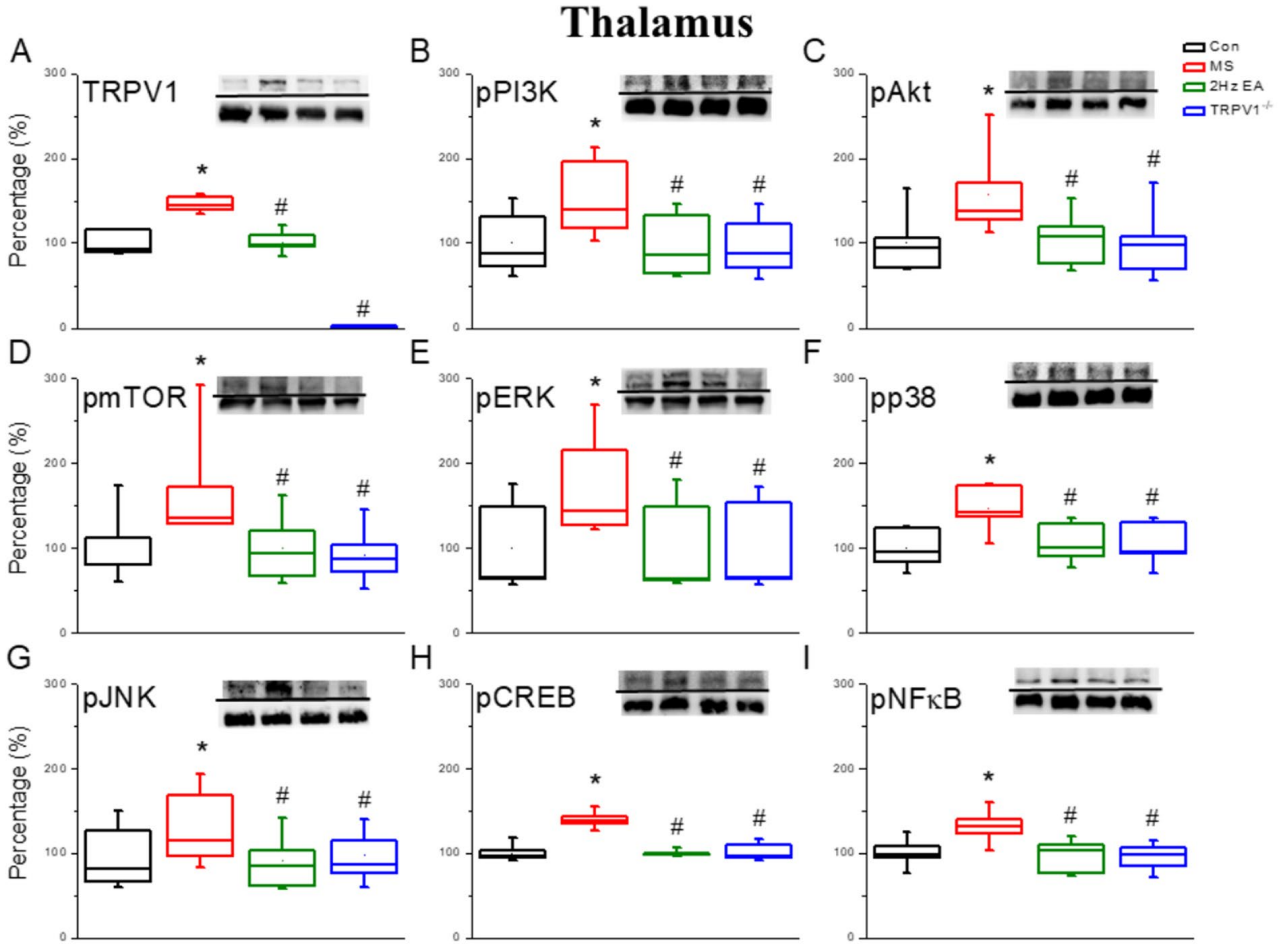
Expression levels of TRPV1-associated signaling pathway in a mouse thalamus. (**A**) TRPV1, (**B**) pPI3K, (**C**) pAKT, (**D**) pmTOR, (**E**) pERK, (**F**) pp38, (**G**) pJNK, (**H**) pCREB, and (**I**) pNFκB protein levels in the thalamus from the Con, MS, EA, and TRPV1^−/−^ groups (from left to right). Con = Control; MS = Motion sickness; EA = 2-Hz electroacupuncture; TRPV1^−/−^, TRPV1 gene deletion. ^*^p < 0.05 vs. Con. ^#^*p* < 0.05 vs. MS group. Western blot bands at the top of the figure show cropped target proteins, and the bands at the bottom show cropped internal controls (β-actin or α-tubulin).

Next, we investigated protein expression levels in the thalamus to examine the effects of EA at acupoint PC6 on the reduction of MS response and to explore the mechanism of MS, focusing specifically on TRPV1 and related molecules. We used Western blotting to observe protein levels and then evaluated protein density. TRPV1 protein level was significantly increased after the MS stimulation phase in the MS group (Fig. 2A; 146.85 ± 3.65, *p* < 0.05) compared with that in the control group. In contrast, it was significantly decreased in the EA group (Fig. 2A; 101.8 ± 5.03, *p* < 0.05) and even more so in the TRPV1^−/−^ group (Fig. 2A; 18.31 ± 2.17, *p* < 0.05) compared with that in the MS group. Furthermore, we observed that there was a similarity in the TRPV1 expression tendency of pPI3K (Fig. 2B; 151.69 ± 17.85, *p* < 0.05) and pAKT (Fig. 2C; 157.55 ± 17.11, *p* < 0.05) compared with that in the control group. The data showed that they were significantly increased in the MS group compared with that in the other groups, whereas they were significantly reduced in the EA group (Fig. 2B and C; 97.52 ± 14.25; 105.89 ± 10.6, *p* < 0.05, respectively) compared with that in the MS and TRPV1^−/−^ groups (Fig. 2B and C; 97.51 ± 13.17; 100.31 ± 13.82, *p* < 0.05, respectively) compared with that in the MS group. We further observed that protein density was significantly increased after the MS stimulation phase in the MS group (Fig. 2D; 168.39 ± 25.76, *p* < 0.05) compared with that in the control group. We also observed a significantly reduced protein density after EA treatment in the EA group (Fig. 2D; 100.38 ± 15.5, *p* < 0.05) and the TRPV1^−/−^ group (Fig. 2D; 92.25 ± 12.88, *p* < 0.05), which lacked the TRPV1 protein, compared with that in the MS group.

Next in the cascade are three major MAPK groups, whose results compared with the control group were as follows: pERK (Fig. 2E; 136.76 ± 18.38, *p* < 0.05); pp38 (Fig. 2F; 147.48 ± 10.67, *p* < 0.05); and pJNK (Fig. 2G; 173.12 ± 19.86, *p* < 0.05), which is rather similar to TRPV1 result propensity (Fig. 2E and G, 100.01 ± 14.62; 99.99 ± 8.89; 99.99 ± 17.18, respectively, *p* < 0.05) compared with the MS group. Furthermore, we observed that it was significantly reduced in the EA group (Fig. 2E and G; 95.32 ± 9.95; 105.72 ± 9.32; 100.91 ± 17.89, respectively, p < 0.05) compared with that in the MS and TRPV1^−/−^ groups (Fig. 2E and G; 95.39 ± 9.91; 105.17 ± 9.97; 100.74 ± 17.14, respectively, *p* < 0.05) compared with the MS group.

Finally, we investigated the downregulation of this mechanism, pCREB and pNFκB, which are in the nucleus of cells and are associated with the transcriptional pathway. After MS stimulation, we observed significant higher levels of pCREB (Fig. 2H; 139.61 ± 3.73, *p* < 0.05) and pNFκB (Fig. 2I; 131.91 ± 7.59, *p* < 0.05) compared with those in the control group. We also observed a significant increase in protein levels in the MS group, which was significantly decreased in the EA group (Fig. 2H and I; 99.46 ± 1.34; 97.98 ± 7.6, respectively, *p* < 0.05) and TRPV1^−/−^ group (Fig. 2H and I; 101.3 ± 3.84; 95.99 ± 6.29, respectively, *p* < 0.05) compared with that in the MS group.

**Figure 3:**
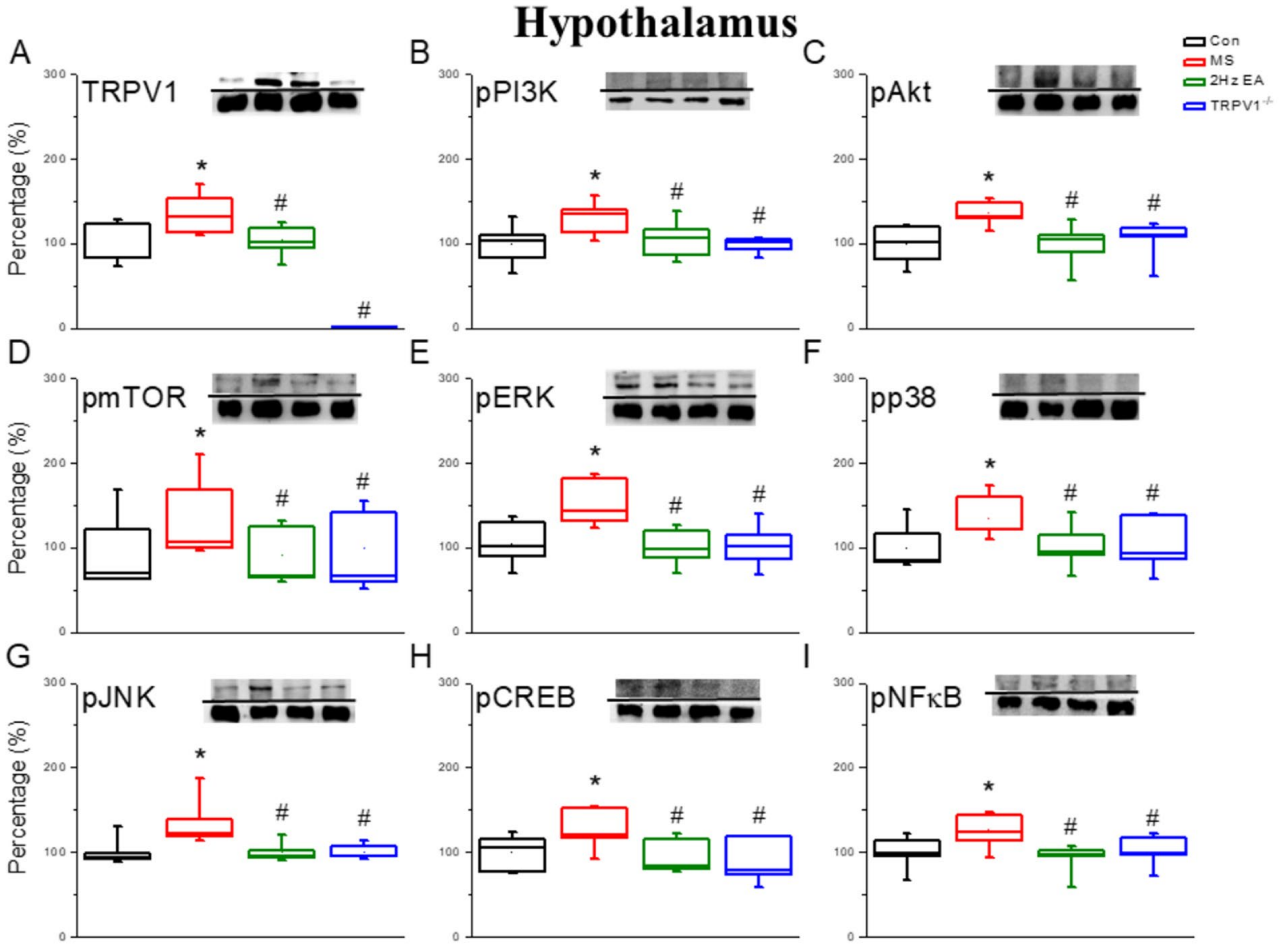
Expression levels of TRPV1-associated signaling pathway in a mouse hypothalamus. (**A**) TRPV1, (**B**) pPI3K, (**C**) pAKT, (**D**) pmTOR, (**E**) pERK, (**F**) pp38, (**G**) pJNK, (**H**) pCREB, and (**I**) pNFκB protein levels in the hypothalamus from the Con, MS, EA, and TRPV1^−/−^ groups (from left to right). Con = Control; MS = Motion sickness; EA = 2-Hz electroacupuncture; TRPV1^−/−^, TRPV1 gene deletion. ^*^p < 0.05 vs. Con. ^#^*p* < 0.05 vs. MS group. Western blot bands at the top show cropped target proteins, and the bands at the bottom show cropped internal controls (β-actin or α-tubulin).

Next, we identified TRPV1 and related protein levels in the hypothalamus. Results indicated that TRPV1 protein levels were significantly increased after MS stimulation (Fig. 3A; 135.62 ± 9.42, *p* < 0.05) compared with those in the control group. TRPV1 protein levels were reduced in the EA group (Fig. 3A; 104.68 ± 7.54, *p* < 0.05) and the TRPV1^−/−^ group (Fig. 3A; 1.16 ± 0.1, p < 0.05) compared with those in the MS group. We also found that the levels of pPI3K (Fig. 3B; 131.42 ± 9.96, *p* < 0.05) and pAKT (Fig. 3C; 136.12 ± 5.85, *p* < 0.05) were increased in MS mice compared with those in the control group. The increased levels of the aforementioned proteins were further attenuated in the EA group (Fig. 3B and C; 105.25 ± 8.73; 99.93 ± 10.14, respectively, *p* < 0.05) and the TRPV1^−/−^ group (Fig. 3B and C; 98.67 ± 3.64; 105.65 ± 9.25, respectively, *p* < 0.05) compared with those in the MS group.

**Figure 4:**
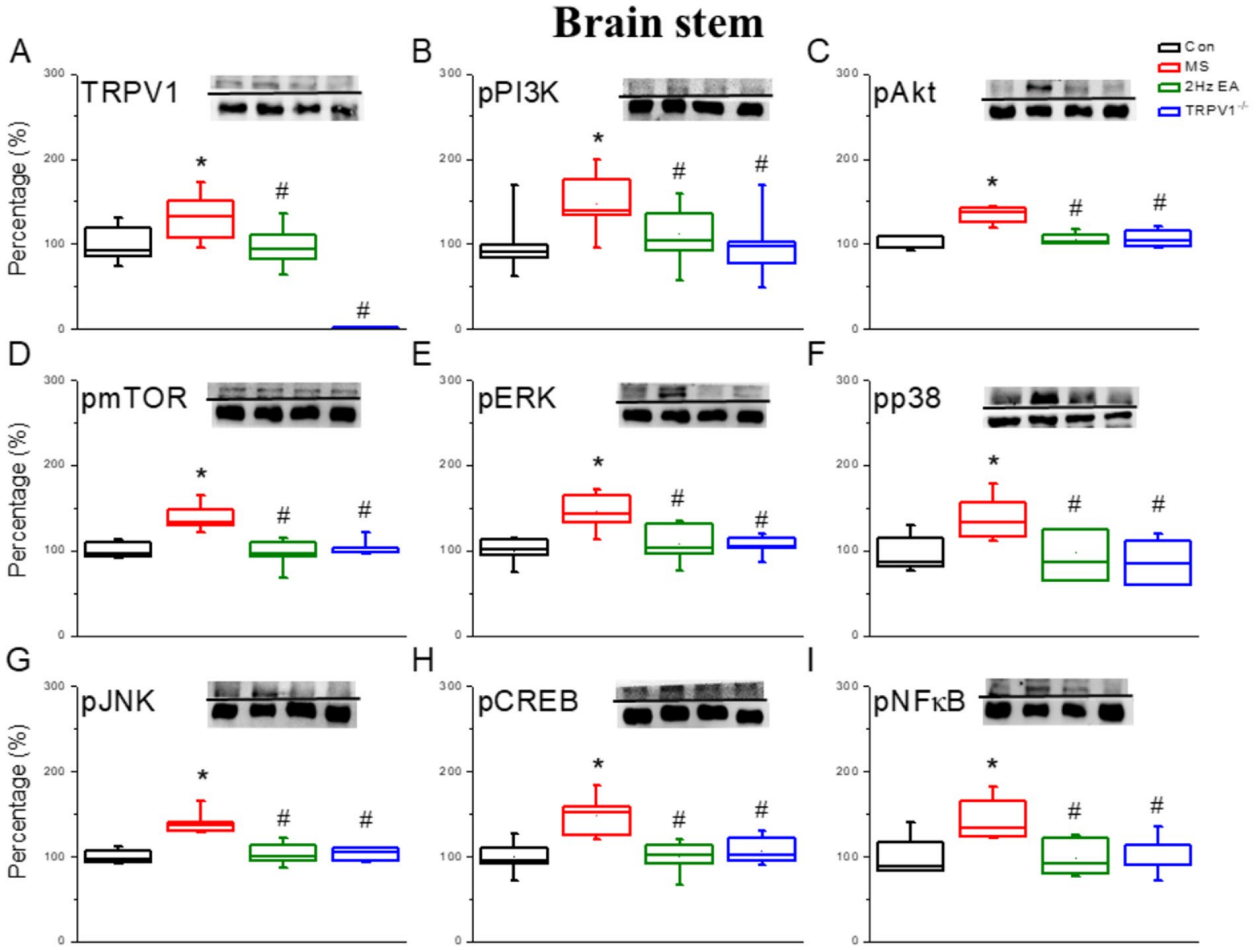
Expression levels of TRPV1-associated signaling pathway in a mouse brain stem. (**A**) TRPV1, (**B**) pPI3K, (**C**) pAKT, (**D**) pmTOR, (**E**) pERK, (**F**) pp38, (**G**) pJNK, (**H**) pCREB, and (**I**) pNFκB protein levels in the brain stem from the Con, MS, EA, and TRPV1^−/−^ groups (from left to right). Con = Control; MS = Motion sickness; EA = 2-Hz electroacupuncture; TRPV1^−/−^, TRPV1 gene deletion. ^*^p < 0.05 vs. Con. ^#^*p* < 0.05 vs. MS group. Western blot bands at the top show cropped target proteins, and the bands at the bottom show cropped internal controls (β-actin or α-tubulin).

Similar results were observed for mTOR levels (Fig. 3D; 138.01 ± 18.54, *p* < 0.05), which were significantly increased after MS induction, compared with those in the control group. mTOR levels significantly decreased in the EA group (Fig. 3D; 91.13 ± 13.02, *p* < 0.05) and the TRPV1^−/−^ group (Fig. 3D, 99.75 ± 18.63, *p* < 0.05) compared with those in the MS group. Furthermore, we found that the levels of pERK (Fig. 3E; 145.57 ± 7.54, *p* < 0.05), pp38 (Fig. 3F; 135.12 ± 10.36, *p* < 0.05), and pJNK (Fig. 3G; 133.73 ± 11.2, *p* < 0.05) were increased after MS induction compared with those in the control group. These increases were reversed in the EA group (Fig. 3E and G; 98.1 ± 6.62; 101.62 ± 10.3; 100.08 ± 4.39, respectively, *p* < 0.05) and the TRPV1^−/−^ group (Fig. 3E and G; 99.3 ± 8.18; 102.98 ± 12.48; 100.84 ± 3.44, respectively, *p* < 0.05) compared with those in the MS group. Moreover, we determined that the levels of pCREB (Fig. 3H; 127.48 ± 9.5, *p* < 0.05) and pNFκB (Fig. 3I; 126.39 ± 8.41, *p* < 0.05) were significantly increased in the MS group compared with those in the control group, and further attenuated in the EA group (Fig. 3H and I, 96.23 ± 7.83; 92.98 ± 7.08, respectively, *p* < 0.05) and TRPV1^−/−^ group (Fig. 3H and I; 91.92 ± 10.42; 102.70 ± 7.23, respectively, *p* < 0.05) compared with those in the MS group. The aforementioned mechanisms were also obtained in the brain stem regions (Fig. 4).

**Figure 5:**
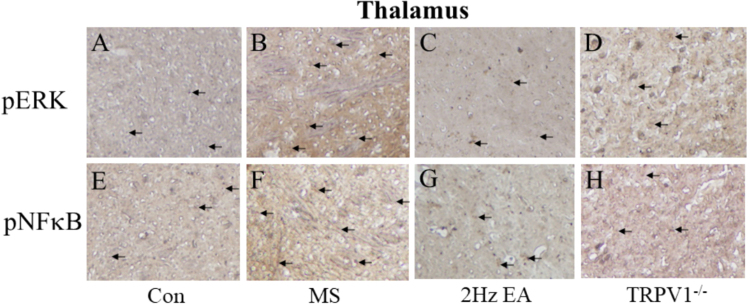
Expression levels of pERK and pNFκB in the thalamus of Con, MS, EA, and TRPV1^−/−^ mice. pERK-positive neurons (brown) in the thalamus of (**A**) Con, (**B**) MS, (**C**) EA, and (**D**) TRPV1^−/−^ mice. pNFκB immuno-positive neurons (brown) in the thalamus of (**E**) Con; (**F**) MS; (**G**) EA; (**H**) and TRPV1^−/−^ mice. Arrows identify immune-positive neurons. Con = Control; MS = Motion sickness; EA = 2-Hz electroacupuncture; TRPV1^−/−^, TRPV1 gene deletion.

**Figure 6:**
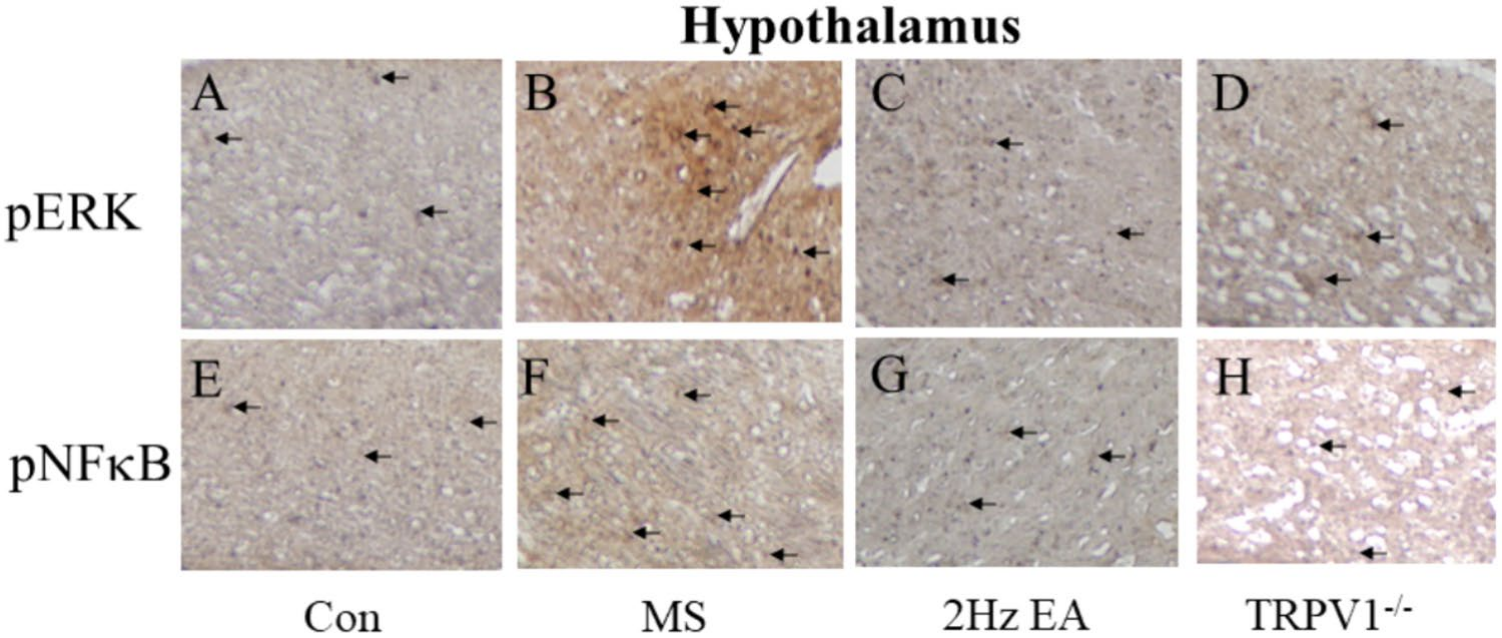
Expression levels of pERK and pNFκB in the hypothalamus of Con, MS, EA, and TRPV1^−/−^ mice. pERK-positive neurons (brown) in the hypothalamus of (**A**) Con, (**B**) MS, (**C**) EA, and (**D**) TRPV1^−/−^ mice. pNFκB immuno-positive neurons (brown) in the thalamus of (**E**) Con, (**F**) MS, (**G**) EA, and (**H**) TRPV1^−/−^ mice. Arrows identify immune-positive neurons. Con = Control; MS = Motion sickness; EA = 2-Hz electroacupuncture; TRPV1^−/−^, TRPV1 gene deletion.

**Figure 7:**
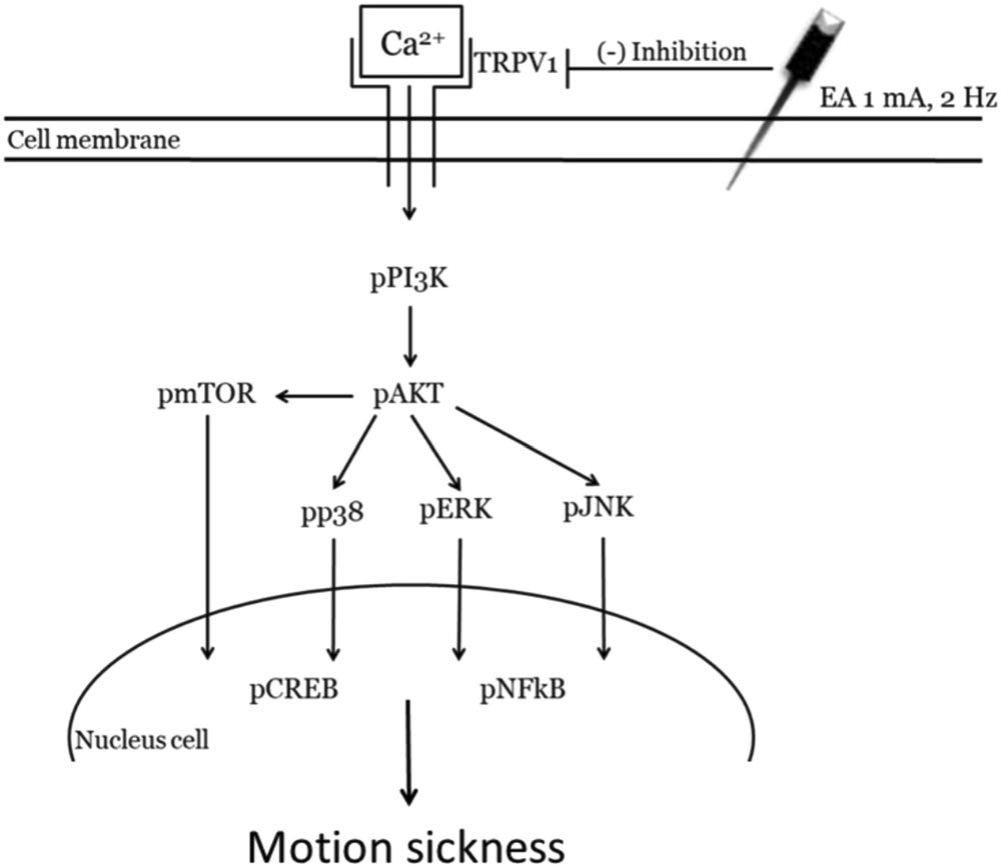
Illustration of detailed mechanisms of TRPV1 in the MS mouse model. We showed that TRPV1 is crucial in the MS mouse model. Activation of TRPV1 increased the expression levels of pPI3K, pAKT, and pmTOR. Furthermore, pERK, pp38, pJNK, pNFκB, and pCREB levels were also increased. The aforementioned phenomena could be attenuated by the EA and TRPV1^−/−^ groups. Con = Control; MS = Motion sickness; EA = 2-Hz electroacupuncture; TRPV1^−/−^, TRPV1 gene deletion.

Finally, using immunohistochemistry, we determined whether TRPV1-related downstream molecules in the thalamus or hypothalamus are required for the MS method. We showed that pERK levels were significantly increased in the thalamus after MS induction (Fig. 5B), whereas this increase was attenuated in the EA and TRPV1^−/−^ groups (Fig. 5C and D). These tendencies were also observed in pNFκB protein levels (Fig. 5E and H). Our data further suggested that pERK was increased in the hypothalamus during MS and that this increase can be reversed by the EA and TRPV1^−/−^ groups (Fig. 6A and D). Similar results were obtained for pNFκB protein levels in the hypothalamus (Fig. 6E and H). Overall, the aforementioned data suggest that TRPV1 and related signaling molecules are involved in MS and are further attenuated by the EA and TRPV1^−/−^ groups (Fig. 7).

## Discussion

It is well known that MS can be stimulated by frequently changing movements and that conflict and neural mismatch from sensory signal inputs initiate several reactions. Although anyone (including healthy individuals) can experience MS, only 5–10% of people are highly susceptible to it. In the present study, kaolin consumption, a measure of MS, was significantly decreased in mice subjected to EA compared to those in whom MS was induced. Meanwhile, there is only a different condition that the EA group was obtained EA after MS stimulation shows the effectiveness of EA treatment for MS reactions. Moreover, kaolin consumption between the MS and TRPV1^−/−^ groups, which received MS stimulation, were significantly different, which implies that provoking MS did not affect mice lacking the TRPV1 receptor. This finding strongly supports the presence of a relationship between TRPV1 and the MS mechanism, suggesting that TRPV1 is one condition that initiates the MS response.

Recent articles have reported an association between MS and histamine-releasing (anti-histamine) medicine, which is the most popular MS treatment in Western medicine^13,35^. Wang *et al*. reported a relationship between MS and the GABA receptor, NMDA signaling focused on habituation behavior without treatment^36^. Furthermore, TRPV1 was reported to be involved in EA for gastric motility, which participates in the major signs and symptoms of MS, and revealed significantly increased inhibition of gastric motility^37^. TRPV1 is a calcium ion channel located in many areas of the body, including the thalamus, responds with diverse stimulating factors, and functions as a part of the MAPK pathway. The association between vomiting and TRPV1 has been examined under the conditions of Western medicine^38^. Results of the present study indicated that TRPV1 and related molecules had similar tendencies in the four groups examined, with the highest protein levels in the MS group and significantly lower levels in the EA and TRPV1^−/−^ groups. Accordingly, we suggest that the TRPV1 signaling cascade is crucial to the MS mechanism.

Acupoint PC6 propels *qi* through the meridian of the pericardium to the heart, in line with the spinal segments C6-T1. Previous research suggested a possible association between the pericardium meridian and the autonomic and central nervous systems^39,40^. A scientific description of the term *qi* is best understood as a releasing of endogenous neurotransmitters in response to stimulation^41^. Besides MS inducing avoidance and sleeping, no other remedy is effective for everyone; therefore, acupuncture is an interesting treatment for MS. This technique can be easily used in any situation, not only in the form of acupuncture or EA but also as acupressure, which was found to effectively relieve the MS response. TRPV1 receptors are non-selective cation channels that function in the MAPK pathway, which is a major communication pathway between extracellular stimuli and intracellular compartments. However, the signals from vestibular organs in the inner ear cascade to the thalamus for MS induction^42^.

We used a mouse model in order to exclude the psychological factors related to MS and EA. To the best of our knowledge, no previous study has determined the effect and/or association between EA and the neural mechanism of MS. The present study focused on the change in protein density in the thalamus, as several thalamic regions are centers of multisensory information processing from visual, otoliths, semicircular canals, and somatosensory stimuli^42^. This sensory cascade to the hypothalamus and brain stem represents an important MS brain response. The thalamus was also implicated in the responsive change when subjects were stimulated at acupoint PC6^43^. In addition, the TRPV1 is a calcium ion channel that responds to several chemical ligands and is located in numerous areas of the body (including the thalamus), which are associated with MS. Consequently, it was hypothesized that the EA treatment would reduce the symptoms of MS via the TRPV1 pathway.

In summary, our results suggest the significant associations between EA stimulation and the MS response via the TRPV channels and related molecular responses. This modulation was demonstrated by kaolin consumption, TRPV1 protein level, and other protein levels involved in the MAPK signaling pathway. Our results also revealed a parallel tendency in which the aforementioned outcomes were significantly increased in the MS group and reduced in the EA and TRPV1^−/−^ groups. We conclude that the expression levels of TRPV1, pPI3K, pAKT, pmTOR, pERK, pp38, pJNK, pCREB, and pNFκB play important roles in the MS mechanism and that the MS response can be alleviated either by electroacupuncturing at acupoint PC6 or by deleting the TRPV1 gene in mice.

Future research could be directed in furthering the evidence of the mechanism of action observed in TRPV1 receptors and associated pathways of MS responses. Identifying a consistently reliable TRPV1 antagonist is paramount in developing effective remedies for MS. Both Western and Chinese Medicine approaches can be scientifically explored in order to optimize potential health care solutions for the public.

## Methods

### Experimental animals

Male 8- to 12-week-old C57BL/6 mice weighing 23–28 g were purchased from BioLasco Taiwan Ltd. (Yilan, Taiwan). The animals were housed individually in home cages (13 × 18.8 × 29.5 cm) under a 12:12-h light–dark cycle (8:00 a.m. to 8:00 *p*.m.) with free access to standard mouse chow (Lab Diet), kaolin (a clay mineral), and water over a 3-day adaptation period before initiation of the experiment. The study was approved by the Institute of Animal Care and Use Committee of China Medical University (Permit no. 2016–061), Taiwan, following the Guide for the Use of Laboratory Animals (National Academy Press). Subjects were randomly divided into four groups of 6 mice each: (1) control group, (2) MS group, (3) EA group, and (4) TRPV1 knockout group (TRPV1^−/−^), forming a sample of 24 mice. Mice were sacrificed on Day 5 of the experiment. Efforts were made to minimize the number of animals used and their sufferings.

### Rotation device and procedure

To provoke MS, subjects from the MS, EA, and TRPV1^−/−^ groups were separately placed in individual cages (9.5 × 11 × 6.5 cm) whose lid was attached to a turntable suspended 3 cm from its axis and rotated at a velocity of 80 rpm continuously for 40 min. The turntable began rotating on a 21-s cycle, starting with a clockwise rotation for 10 s followed by a counterclockwise rotation for 10 s; the two directions were separated by a pause of less than 1 s. The MS stimulation procedure was performed for 4 days, from 9:00 to 10:00 a.m., and animals were then returned to their individual observation home cages. Subjects in the control group were excluded from this MS stimulation phase.

### Electroacupuncture

Mice in the EA group received EA treatment on the bilateral side of acupoint PC6 after the MS stimulation phase. Sterile 0.2 × 13-in needles (0.5 in, 32 G; Yu Kuang Chem. Ind. Corp., Taiwan) were used to apply EA. For reference, in humans, acupoint PC6 is located in the interosseal muscles between the radius and the ulna bones of the distal medial thoracic limb, 3 mm superior to the wrist joint. The electric stimulator was attached to the needles at an amplitude of 1 mA and a frequency of 2 Hz with a pulse width of 150 µs for 15 min. The intensity was required to be strong enough to elicit slight twitches of the limbs.

### Kaolin, mice chow, and water preparation

Kaolin (Sigma-Aldrich Co., USA) was mixed with 1% acacia gum (Sigma-Aldrich Co.) (w/w) in water to form a thick paste. Pellets were then molded into the same shape as those of standard mouse chow and dried at room temperature. Kaolin pellets were provided in a separate container next to the food container in each mouse’s home cage. Kaolin and mice chow pellets were collected, dried, weighed, refilled, and replaced daily, and water was measured regularly between 8:00 and 9:00 a.m. throughout the experimental period to obtain values of kaolin, food, and water consumption.

### Western blotting analysis

In this experiment, animals were anesthetized with isoflurane by inhalation processes. After each mouse was sacrificed, the thalamus was immediately dissected and frozen in ice before being stored at −80 °C. Total proteins were prepared through abrasion and were lysed using a solution of 50 mM Tris–HCl (pH 7.4), 250 mM NaCl, 1% NP-40, 5 mM EDTA, 50 mM NaF, 1 mM Na_3_VO_4_, 0.02% NaN_3_, and 1 × protease inhibitor cocktail (Amresco, Solon, OH, USA) before being centrifuged. Proteins from each sample were loaded on 8% Tris-glycine-SDS gel electrophoresis and transferred onto polyvinylidene difluoride membranes. Afterward, they were blocked with 5% non-fat milk in TBST buffer [10 mM Tris (pH 7.5), 100 mM NaCl, and 0.1% Tween 20] and incubated for 1 h at room temperature with primary antibodies in TBST with 1% bovine serum albumin. Peroxidase-conjugated anti-mouse and anti-rabbit antibody (1:5,000) was used as a secondary antibody. Protein bands on the membranes were visualized using an enhanced chemiluminescent substrate kit (PIERCE) with LAS-3000 Fujifilm (Fuji Photo Film Co. Ltd). Image densities of specific bands were quantified using ImageJ software (National Institutes of Health, Bethesda, MD, USA).

### IHC staining

The mice were anesthetized with 2% isoflurane and then intracardially perfused with saline. The brains were removed and postfixed in the same fixative overnight at 4 °C. After washing with PBS, the brains were move to a 30% sucrose solution in 0.01 M PBS for cryoprotection, and sagittal sections containing the thalamus and hypothalamus area were cut into 16-ſm-thick slices through cryosectioning. The slices were incubated for 10 min at room temperature with 10% normal goat serum in PBS to reduce nonspecific binding. The sections were further incubated at 4 °C overnight with PBS containing the primary antibodies to pERK (Cell Signaling, USA; 1:1000) and pNFκB (1:1000; Abcam, UK). The sections were subsequently incubated with the biotinylated-conjugated secondary antibody (diluted at 1:200; Vector, Burlingame, CA 94010, USA) for 10 min at room temperature, followed by incubation with the avidin–horseradish peroxidase complex (ABC kit, Genemed, USA). The sections were finally visualized using 3,3′-diaminobenzidine as the chromogen. During the incubation steps, the sections were washed with PBS three times for 10 min per cycle. The stained thalamus and hypopthalamus slices were sealed under the coverslips, and then examined for the presence of immune-positive neurons using a microscope (Olympus, BX-51, Japan).

### Data analysis

Statistical analysis was performed using SPSS 12. Statistical comparisons were evaluated using analysis of variance followed by Tukey’s post hoc test. All data presented were expressed as mean ± standard error of the mean. A value of *p* < 0.05 was considered statistically significant.

## Electronic supplementary material

Supplementary information
