# Supplementary material for: Targeting TRPV1 to relieve motion sickness symptoms in mice by electroacupuncture and gene deletion
Source: Sci Rep. 2018 Jul 9;8:10365. doi: 10.1038/s41598-018-23793-6 (PMC6037734; doi:10.1038/s41598-018-23793-6)
Supplement: Supplementary file 1 — Supplementary information [file 41598_2018_23793_MOESM1_ESM.pdf]

Targeting TRPV1 to relieve motion sickness symptoms in mice by electroacupuncture and gene deletion

Chanya Inprasit[1*], Yi-Wen Lin[2,3*], Chun-Ping Huang[2], Shu-Yih Wu[4], Ching-Liang Hsieh[2,3,5,6 #]

[1]College of Chinese Medicine, Graduate Institute of Acupuncture Science International Master Program, China Medical University, Taichung 40402, Taiwan

[2]Research Center for Chinese Medicine & Acupuncture, China Medical University, Taichung 40402, Taiwan

[3]College of Chinese Medicine, Graduate Institute of Acupuncture Science, China Medical University, Taichung 40402, Taiwan

[4]Department of Rehabilitation Medicine, Mackay Memorial Hospital, Taipei, Taiwan

[5]College of Chinese Medicine, Graduate Institute of Integrated Medicine, China Medical University, Taichung 40402, Taiwan

[6]Department of Chinese Medicine, China Medical University Hospital, Taichung 40402, Taiwan

[*]The authors contributed equally to this work.

[#]Correspondence should be addressed to Dr. Ching-Liang Hsieh, Graduate Institute of Integrated Medicine, China Medical University. 91 Hsueh-Shih Road, Taichung 40402, Taiwan

Tel: +886-4-22053366 (ext. 3500)

Fax: +886-4-22037690

E-mail: clhsieh@mail.cmuh.org.tw

Supplementary information

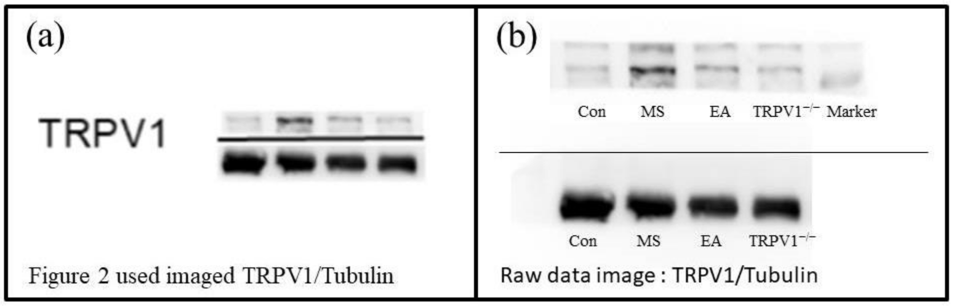

Fig. S1 Expression level of TRPV1 in a mouse thalamus. (a) The figure 2A used image; (b) The original raw data images of TRPV1 protein and internal control. There are 4 lanes of TRPV1 protein, control group, MS group, EA group and TRPV1$^{-/-}$ group. The blot results present a significant increase of TRPV1 expression in MS group compared with the other groups, however, this increase is statistically decreased in the EA group as compared to the base line of the control group.

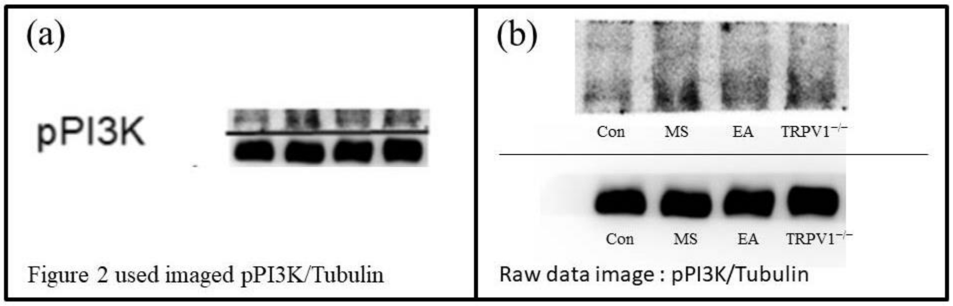

Fig. S2 Expression level of pPI3K in a mouse thalamus. (a) The figure 2B used image; (b) The original raw data images of pPI3K protein and internal control. There are 4 lanes of pPI3K protein, control group, MS group, EA group and TRPV1$^{-/-}$ group. The blot results present a significant increase of pPI3K expression in MS group compared with the other groups, however, this increase is statistically decreased in the EA group as compared to the base line of the control group.

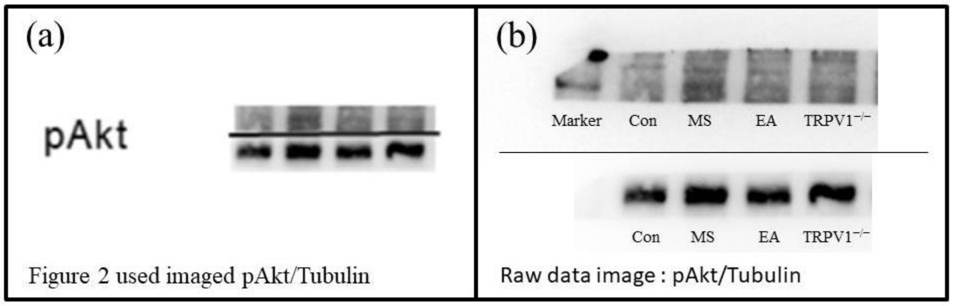

Fig. S3 Expression level of pAkt in a mouse thalamus. (a) The figure 2C used image; (b) The original raw data images of pAkt protein and internal control. There are 4 lanes of pAkt protein, control group, MS group, EA group and TRPV1$^{-/-}$ group. The blot results present a significant increase of pAkt expression in MS group compared with the other groups, however, this increase is statistically decreased in the EA group as compared to the base line of the control group.

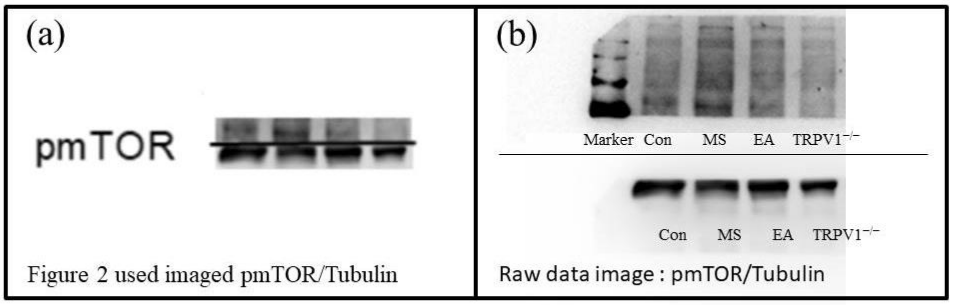

Fig. S4 Expression level of pmTOR in a mouse thalamus. (a) The figure 2D used image; (b) The original raw data images of pmTOR protein and internal control. There are 4 lanes of pmTOR protein, control group, MS group, EA group and TRPV1$^{-/-}$ group. The blot results present a significant increase of pmTOR expression in MS group compared with the other groups, however, this increase is statistically decreased in the EA group as compared to the base line of the control group.

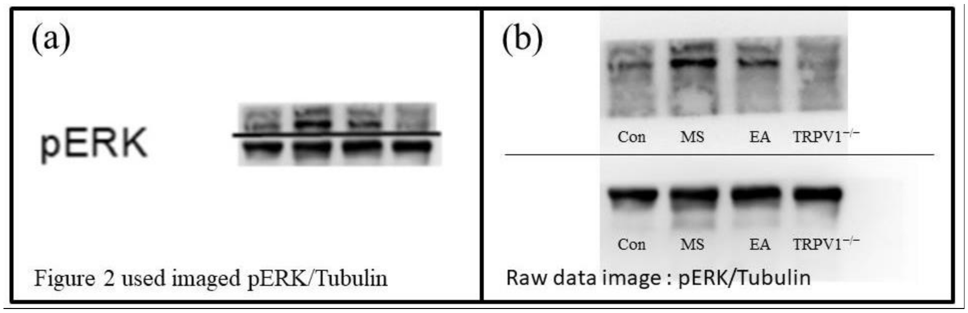

Fig. S5 Expression level of pERK in a mouse thalamus. (a) The figure 2E used image; (b) The original raw data images of pERK protein and internal control. There are 4 lanes of pERK protein, control group, MS group, EA group and TRPV1$^{-/-}$ group. The blot results present a significant increase of pERK expression in MS group compared with the other groups, however, this increase is statistically decreased in the EA group as compared to the base line of the control group.

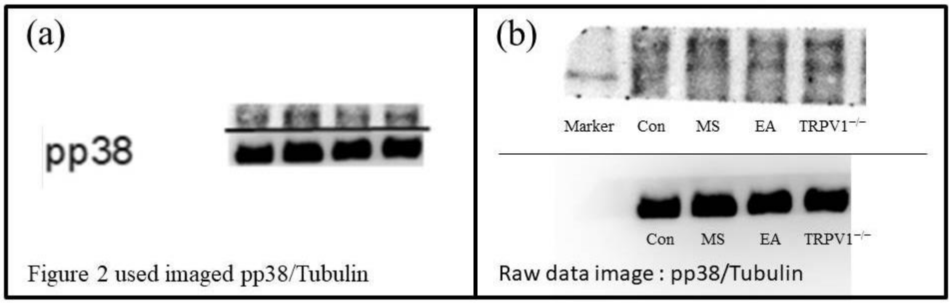

Fig. S6 Expression level of pp38 in a mouse thalamus. (a) The figure 2F used image; (b) The original raw data images of pp38 protein and internal control. There are 4 lanes of pp38 protein, control group, MS group, EA group and TRPV1$^{-/-}$ group. The blot results present a significant increase of pp38 expression in MS group compared with the other groups, however, this increase is statistically decreased in the EA group as compared to the base line of the control group.

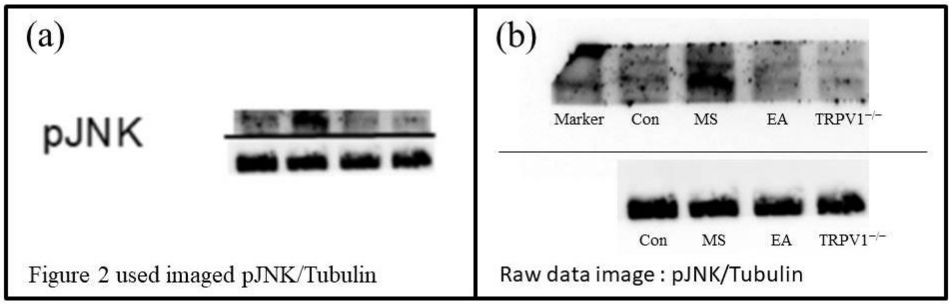

Fig. S7 Expression level of pJNK in a mouse thalamus. (a) The figure 2G used image; (b) The original raw data images of pJNK protein and internal control. There are 4 lanes of pJNK protein, control group, MS group, EA group and TRPV1$^{-/-}$ group. The blot results present a significant increase of pJNK expression in MS group compared with the other groups, however, this increase is statistically decreased in the EA group as compared to the base line of the control group.

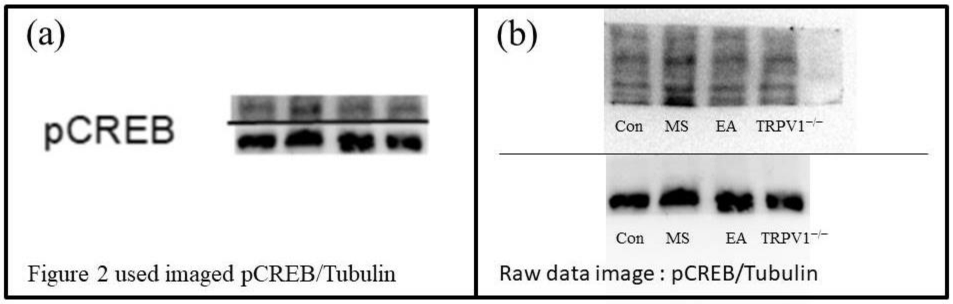

Fig. S8 Expression level of pCREB in a mouse thalamus. (a) The figure 2H used image; (b) The original raw data images of pCREB protein and internal control. There are 4 lanes of pCREB protein, control group, MS group, EA group and TRPV1$^{-/-}$ group. The blot results present a significant increase of pCREB expression in MS group compared with the other groups, however, this increase is statistically decreased in the EA group as compared to the base line of the control group.

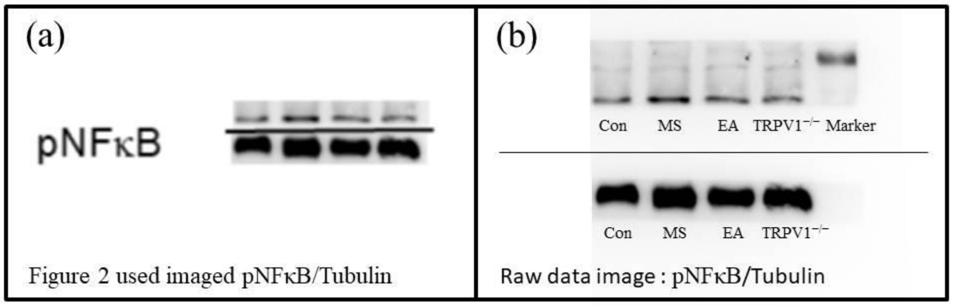

Fig. S9 Expression level of pNFκB in a mouse thalamus. (a) The figure 2I used image; (b) The original raw data images of pNFκB protein and internal control. There are 4 lanes of pNFκB protein, control group, MS group, EA group and TRPV1$^{-/-}$ group. The blot results present a significant increase of pNFκB expression in MS group compared with the other groups, however, this increase is statistically decreased in the EA group as compared to the base line of the control group.

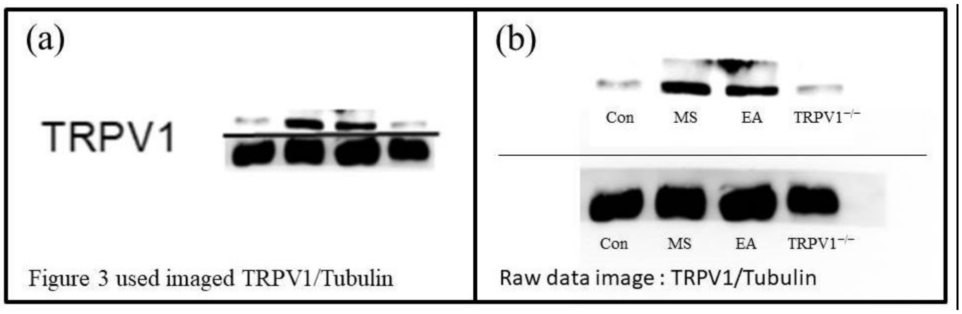

Fig. S10 Expression level of TRPV1 in a mouse hypothalamus. (a) The figure 3A used image; (b) The original raw data images of TRPV1 protein and internal control. There are 4 lanes of TRPV1 protein, control group, MS group, EA group and TRPV1$^{-/-}$ group. The blot results present a significant increase of TRPV1 expression in MS group compared with the other groups, however, this increase is statistically decreased in the EA group as compared to the base line of the control group.

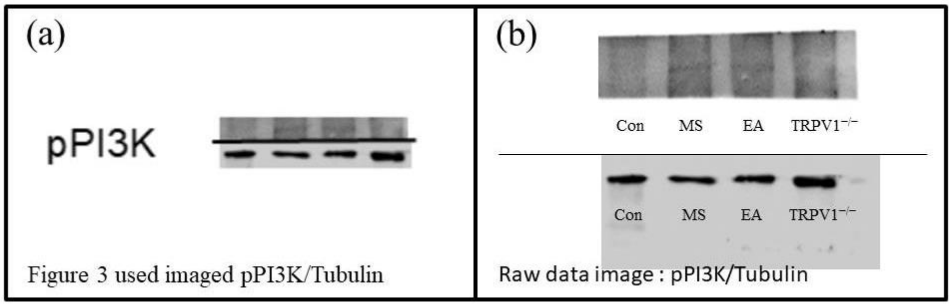

Fig. S11 Expression level of pPI3K in a mouse hypothalamus. (a) The figure 3B used image; (b) The original raw data images of pPI3K protein and internal control. There are 4 lanes of pPI3K protein, control group, MS group, EA group and TRPV1$^{-/-}$ group. The blot results present a significant increase of pPI3K expression in MS group compared with the other groups, however, this increase is statistically decreased in the EA group as compared to the base line of the control group.

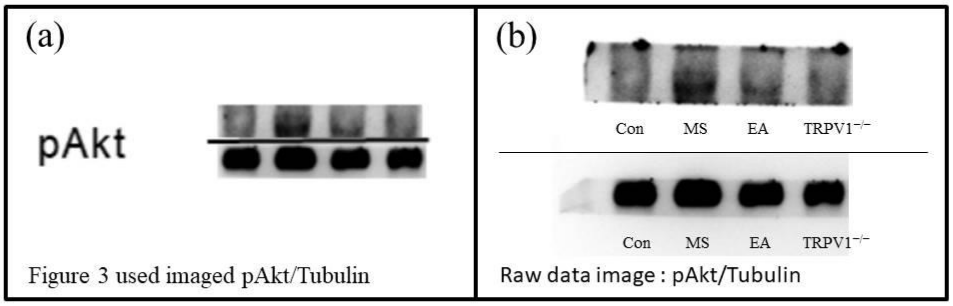

Fig. S12 Expression level of pAkt in a mouse hypothalamus. (a) The figure 3C used image; (b) The original raw data images of pAkt protein and internal control. There are 4 lanes of pAkt protein, control group, MS group, EA group and TRPV1$^{-/-}$ group. The blot results present a significant increase of pAkt expression in MS group compared with the other groups, however, this increase is statistically decreased in the EA group as compared to the base line of the control group.

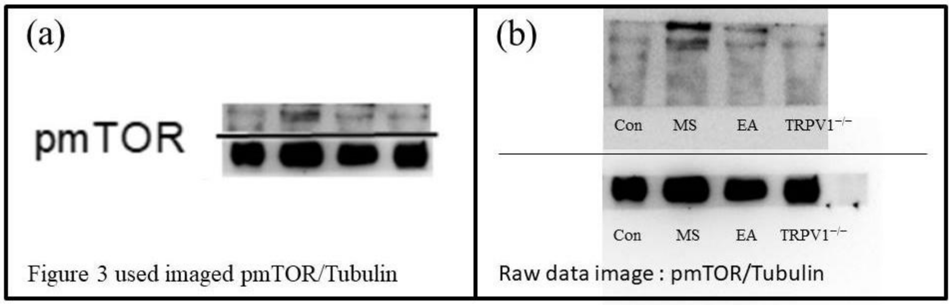

Fig. S13 Expression level of pmTOR in a mouse hypothalamus. (a) The figure 3D used image; (b) The original raw data images of pmTOR protein and internal control. There are 4 lanes of pmTOR protein, control group, MS group, EA group and TRPV1$^{-/-}$ group. The blot results present a significant increase of pmTOR expression in MS group compared with the other groups, however, this increase is statistically decreased in the EA group as compared to the base line of the control group.

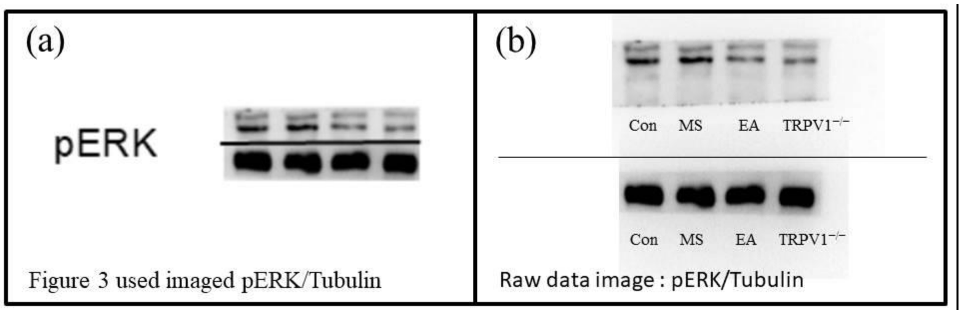

Fig. S14 Expression level of pERK in a mouse hypothalamus. (a) The figure 3E used image; (b) The original raw data images of pERK protein and internal control. There are 4 lanes of pERK protein, control group, MS group, EA group and TRPV1$^{-/-}$ group. The blot results present a significant increase of pERK expression in MS group compared with the other groups, however, this increase is statistically decreased in the EA group as compared to the base line of the control group.

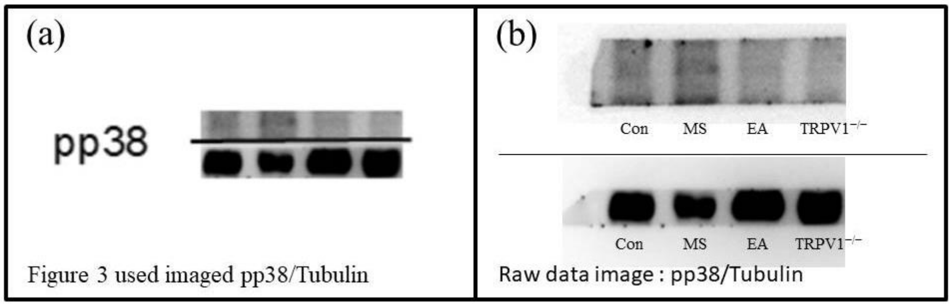

Fig. S15 Expression level of pp38 in a mouse hypothalamus. (a) The figure 3F used image; (b) The original raw data images of pp38 protein and internal control. There are 4 lanes of pp38 protein, control group, MS group, EA group and TRPV1$^{-/-}$ group. The blot results present a significant increase of pp38 expression in MS group compared with the other groups, however, this increase is statistically decreased in the EA group as compared to the base line of the control group.

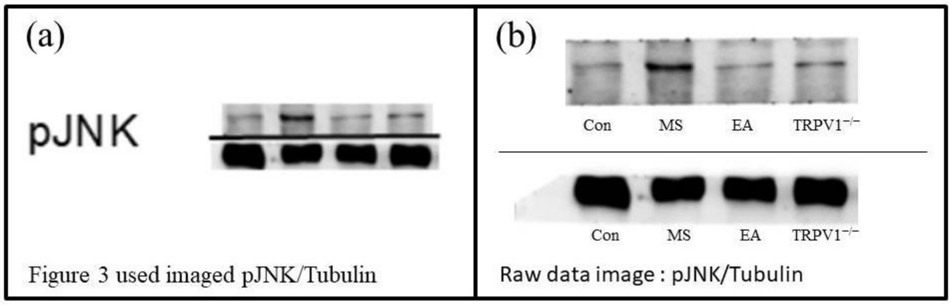

Fig. S16 Expression level of pJNK in a mouse hypothalamus. (a) The figure 3G used image; (b) The original raw data images of pJNK protein and internal control. There are 4 lanes of pJNK protein, control group, MS group, EA group and TRPV1$^{-/-}$ group. The blot results present a significant increase of pJNK expression in MS group compared with the other groups, however, this increase is statistically decreased in the EA group as compared to the base line of the control group.

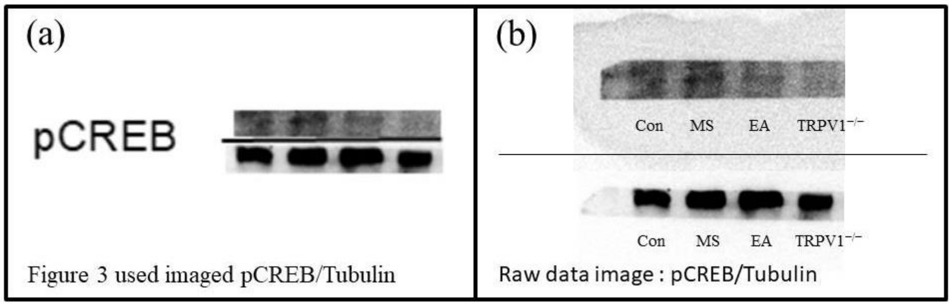

Fig. S17 Expression level of pCREBin a mouse hypothalamus. (a) The figure 3H used image; (b) The original raw data images of pCREB protein and internal control. There are 4 lanes of pCREB protein, control group, MS group, EA group and TRPV1$^{-/-}$ group. The blot results present a significant increase of pCREB expression in MS group compared with the other groups, however, this increase is statistically decreased in the EA group as compared to the base line of the control group.

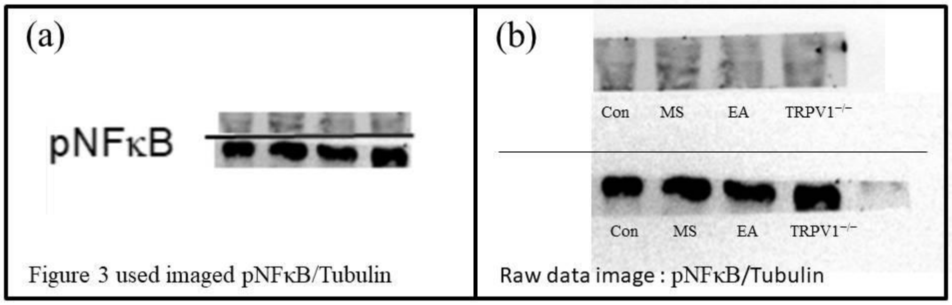

Fig. S18 Expression level of pNFκB in a mouse hypothalamus. (a) The figure 3I used image; (b) The original raw data images of pNFκB protein and internal control. There are 4 lanes of pNFκB protein, control group, MS group, EA group and TRPV1$^{-/-}$ group. The blot results present a significant increase of pNFκB expression in MS group compared with the other groups, however, this increase is statistically decreased in the EA group as compared to the base line of the control group.

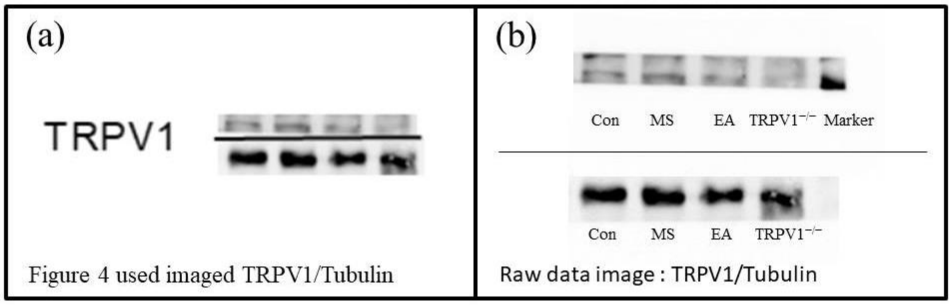

Fig. S19 Expression level of TRPV1 in a mouse brain stem. (a) The figure 4A used image; (b) The original raw data images of TRPV1 protein and internal control. There are 4 lanes of TRPV1 protein, control group, MS group, EA group and TRPV1-/- group. The blot results present a significant increase of TRPV1 expression in MS group compared with the other groups, however, this increase is statistically decreased in the EA group as compared to the base line of the control group.

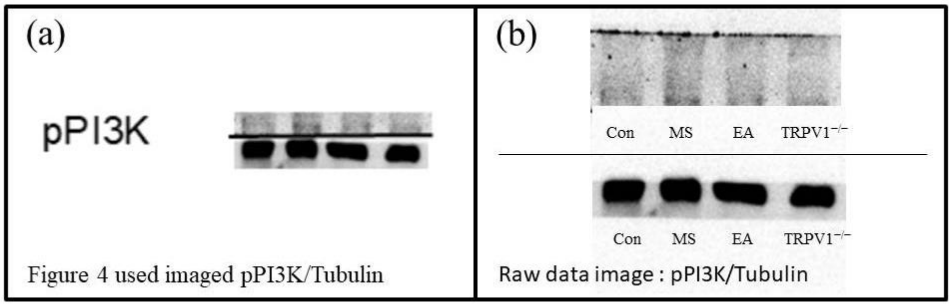

Fig. S20 Expression level of pPI3K in a mouse brain stem. (a) The figure 4B used image; (b) The original raw data images of pPI3K protein and internal control. There are 4 lanes of pPI3K protein, control group, MS group, EA group and TRPV1$^{-/-}$ group. The blot results present a significant increase of pPI3K expression in MS group compared with the other groups, however, this increase is statistically decreased in the EA group as compared to the base line of the control group.

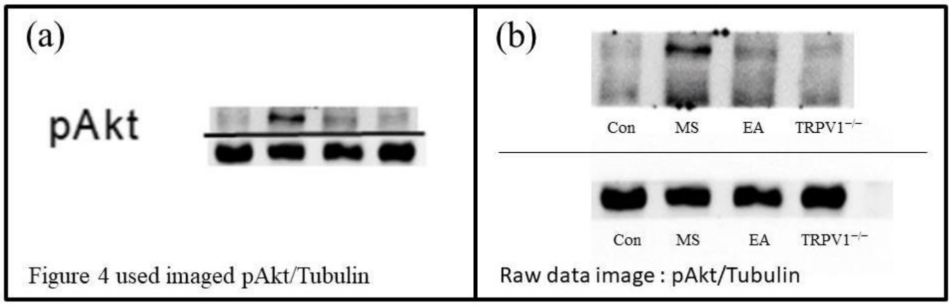

Fig. S21 Expression level of pAkt in a mouse brain stem. (a) The figure 4C used image; (b) The original raw data images of pAkt protein and internal control. There are 4 lanes of pAkt protein, control group, MS group, EA group and TRPV1$^{-/-}$ group. The blot results present a significant increase of pAkt expression in MS group compared with the other groups, however, this increase is statistically decreased in the EA group as compared to the base line of the control group.

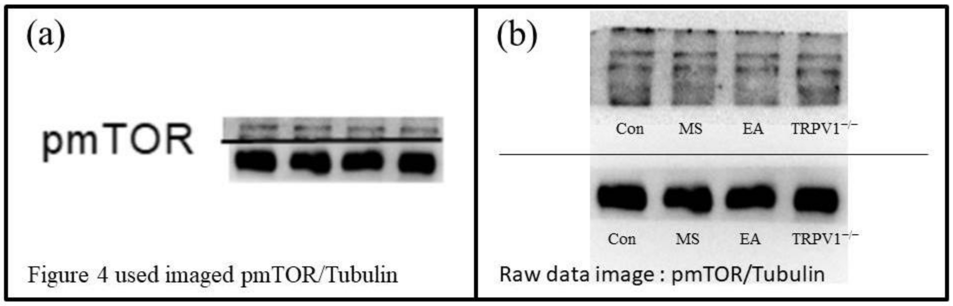

Fig. S22 Expression level of pmTOR in a mouse brain stem. (a) The figure 4D used image; (b) The original raw data images of pmTOR protein and internal control. There are 4 lanes of pmTOR protein, control group, MS group, EA group and TRPV1$^{-/-}$ group. The blot results present a significant increase of pmTOR expression in MS group compared with the other groups, however, this increase is statistically decreased in the EA group as compared to the base line of the control group.

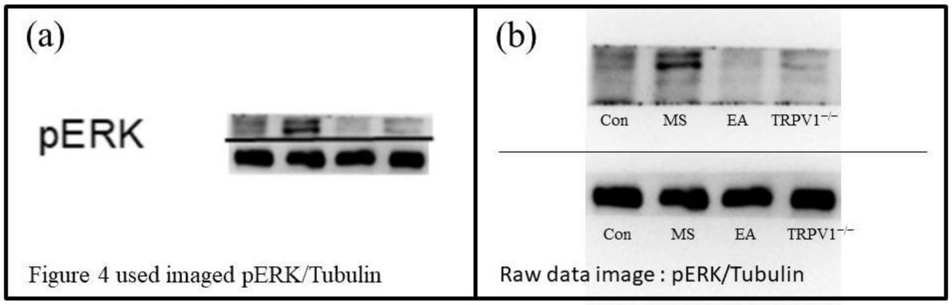

Fig. S23 Expression level of pERK in a mouse brain stem. (a) The figure 4E used image; (b) The original raw data images of pERK protein and internal control. There are 4 lanes of pERK protein, control group, MS group, EA group and TRPV1$^{-/-}$ group. The blot results present a significant increase of pERK expression in MS group compared with the other groups, however, this increase is statistically decreased in the EA group as compared to the base line of the control group.

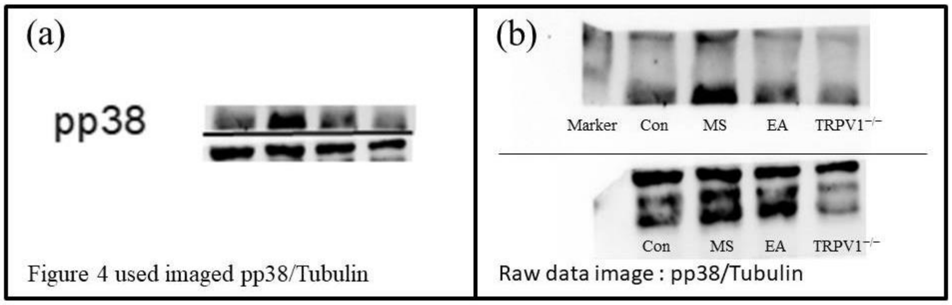

Fig. S24 Expression level of pp38 in a mouse brain stem. (a) The figure 4F used image; (b) The original raw data images of pp38 protein and internal control. There are 4 lanes of pp38 protein, control group, MS group, EA group and TRPV1$^{-/-}$ group. The blot results present a significant increase of pp38 expression in MS group compared with the other groups, however, this increase is statistically decreased in the EA group as compared to the base line of the control group.

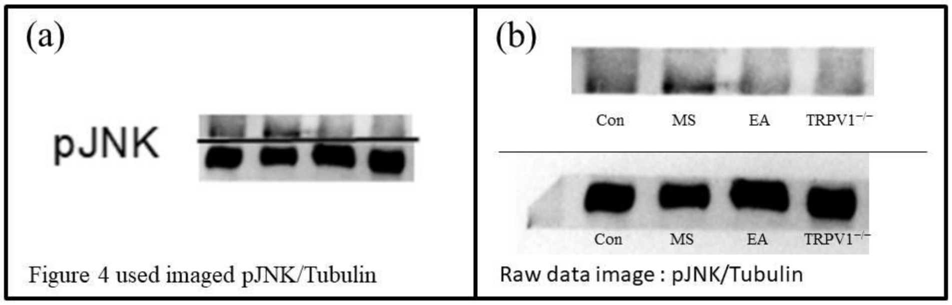

Fig. S25 Expression level of pJNK in a mouse brain stem. (a) The figure 4G used image; (b) The original raw data images of pJNK protein and internal control. There are 4 lanes of pJNK protein, control group, MS group, EA group and TRPV1$^{-/-}$ group. The blot results present a significant increase of pJNK expression in MS group compared with the other groups, however, this increase is statistically decreased in the EA group as compared to the base line of the control group.

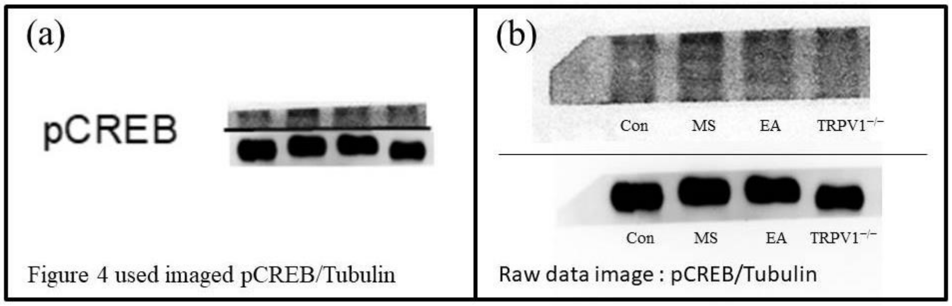

Fig. S26 Expression level of pCREB in a mouse brain stem. (a) The figure 4H used image; (b) The original raw data images of pCREB protein and internal control. There are 4 lanes of pCREB protein, control group, MS group, EA group and TRPV1$^{-/-}$ group. The blot results present a significant increase of pCREB expression in MS group compared with the other groups, however, this increase is statistically decreased in the EA group as compared to the base line of the control group.

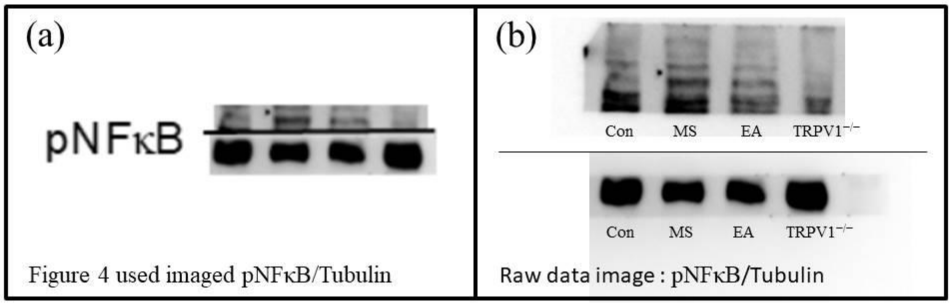

Fig. S27 Expression level of pNFκB in a mouse brain stem. (a) The figure 4I used image; (b) The original raw data images of pNFκB protein and internal control. There are 4 lanes of pNFκB protein, control group, MS group, EA group and TRPV1$^{-/-}$ group. The blot results present a significant increase of pNFκB expression in MS group compared with the other groups, however, this increase is statistically decreased in the EA group as compared to the base line of the control group.
